# Seasonal variation of behavioural thermoregulation in a fossorial salamander (*Ambystoma maculatum*)

**DOI:** 10.1098/rsos.240537

**Published:** 2024-09-04

**Authors:** Danilo Giacometti, Glenn J. Tattersall

**Affiliations:** ^1^ Department of Biological Sciences, Brock University, St Catharines, Ontario L2S 3A1, Canada

**Keywords:** amphibian, body temperature, ectotherm, fossorial, seasonality, thermal biology

## Abstract

Temperature seasonality plays a pivotal role in shaping the thermal biology of ectotherms. However, we still have a limited understanding of how ectotherms maintain thermal balance in the face of varying temperatures, especially in fossorial species. Due to thermal buffering underground, thermal ecology theory predicts relaxed selection pressure over thermoregulation in fossorial ectotherms. As a result, fossorial ectotherms typically show low thermoregulatory precision and low evidence of thermotactic behaviours in laboratory thermal gradients. Here, we evaluated how temperature selection (*T*_sel_) and associated behaviours differed between seasons in a fossorial amphibian, the spotted salamander (*Ambystoma maculatum*). By comparing thermoregulatory parameters between the active and overwintering seasons, we show that *A. maculatum* engages in active behavioural thermoregulation despite being fossorial. In both seasons, *T*_sel_ was consistently offset higher than acclimatization temperatures. Thermoregulation differed between seasons, with salamanders having higher *T*_sel_ and showing greater evidence of thermophilic behaviours in the active compared with the overwintering season. Additionally, our work lends support to experimental assumptions commonly made but seldom tested in thermal biology studies. Ultimately, our study demonstrates that the combination of careful behavioural and thermal biology measurements is a necessary step to better understand the mechanisms that underlie body temperature control in amphibians.

## 1. Introduction

Ectotherms depend on external sources of heat to regulate body temperature (*T*_b_) within favourable values because of their relatively low ability to generate and retain endogenous heat [1]. Shifts in environmental temperatures affect organismal processes of ectotherms at all levels of organization, from energy turnover [2] to locomotion [3]. To maintain thermal balance, an ectotherm must sense and process the range and amplitude of environmental temperature variation. Coordinated physiological and behavioural processes are then put in action to modulate heat transfers between the organism and its surroundings [4]. In lizards, for instance, an increase in heart rate and peripheral blood flow have been shown to improve the delivery of warmed blood to the core during basking [5,6]. In amphibians, however, the study of thermoregulation is complicated by high rates of evaporative cooling due to elevated cutaneous water loss [7,8]. Indeed, amphibians have a relatively thin epidermis that allows for oxygen uptake and carbon dioxide excretion at the expense of high rates of cutaneous water loss [9]. Theoretical and empirical work suggested that amphibians prioritize hydroregulation over thermoregulation [10,11], which led some to posit that responses to temperature in amphibians are primarily hydroregulatory instead of thermoregulatory (reviewed in Navas *et al*. [12]). However, amphibians are known to engage in behavioural thermoregulation both in the field [13,14] and in the laboratory [15,16]. Moreover, some amphibians actively thermoregulate even in instances of obligate cutaneous respiration, as seen in lungless salamanders [17] and in frogs that overwinter under the ice of frozen lakes [18]. As such, a balance between thermoregulation and hydroregulation is the most likely scenario in amphibians [7,19]. Despite the evidence challenging the notion that amphibians may show little motivation to thermoregulate due to thermal constraints over desiccation [20], formal assessments of the behaviours involved in amphibian thermoregulation are lacking [15], especially in the context of seasonality.

Long-lived organisms experience seasonal shifts in climate multiple times over their lives [21], and seasonality has been shown to exert a strong selection pressure over the thermal biology of ectotherms [22,23]. To cope with seasonality, some species may enter dormancy during periods of low environmental temperatures or drought [24,25]. While dormant, animals typically do not feed and remain mostly inactive to save energy until favourable climatic conditions return [26]. Other species, however, may be naturally selected to express flexible phenotypes that aid with maintaining performance and thermal balance in the face of varying environmental conditions [27]. In this case, seasonal changes in environmental parameters provide a signal for a shift in the thermal dependence of reaction norms [28] and thermoregulatory drive (*sensu* [15]), thereby eliciting acclimatization. Consequently, ectotherms from seasonal habitats typically exhibit different thermoregulatory behaviours and target different *T*_b_*s* between seasons, being able to sustain activity across a wide range of *T*_b_*s* [29–31]. Thus, by documenting seasonal changes in thermoregulatory parameters, one may gain insight into the behavioural processes that underlie *T*_b_ control in ectotherms [23]. Among vertebrate ectotherms, inquiry into the effect of seasonality over thermoregulation is taxonomically biased toward lizards (e.g. [23,32,33]) . Truly, despite the relatively large number of studies using amphibians as model systems in thermal biology research (e.g. [34,35]), the extent to which thermal traits are capable of seasonal acclimation (i.e. response to a single environmental parameter) or acclimatization (i.e. response to multiple environmental parameters) remains unclear [15] (but see [36]). Moreover, amphibian thermoregulation is known to vary both inter- and intraspecifically, and according to methodological procedures (e.g. acclimation temperatures, photoperiod regime) [15,35].

Researchers investigating how seasonality affects thermoregulation in amphibians typically maintain animals under contrasting thermal and photoperiod regimes, and then allow the animals to make thermally motivated choices within a laboratory thermal gradient (e.g. [17,37]) . The rationale behind the use of thermal gradients to infer thermoregulation presupposes that thermoregulatory costs are low under laboratory conditions [38], selected body temperatures (*T*_sel_) represent the fundamental thermal niche of an organism [39], and movement within the gradient results from thermotaxis (*sensu* [15,40]). Studies in this group typically document seasonal changes in *T*_sel_ without specifically reporting on thermoregulatory behaviours [15]. However, solely highlighting patterns of *T*_sel_ change does not provide enough information on the behavioural processes that underlie *T*_b_ control. Thus, it is important not to assume *a priori* that any seasonal change in *T*_sel_ occurs due to a shift in the motivation to thermoregulate [23], especially in species that may not use overt behaviours for *T*_b_ control. To overcome this limitation, behavioural observations may be integrated with *T*_sel_ measurements; past information on the study species is key to determine which behaviours should be scored [41]. For instance, basking [42] or postural adjustments [13] may be scored in assays with diurnal frogs, as these behaviours are associated with obtaining body heat via solar radiation in diurnal species [43]. By contrast, micro-habitat selection should be the main thermoregulatory behaviour in species from habitats where temperature is relatively stable [44–46], such as nocturnal and/or fossorial salamanders. For these species, assessing how movement patterns relate to changes in *T*_sel_ should be key to gauge seasonal shifts in the motivation to thermoregulate [15].

In this study, we evaluated the effect of seasonality over behavioural thermoregulation in the spotted salamander (*Ambystoma maculatum*). Despite the large abundance of *A. maculatum* populations in parts of North America [47], the thermal biology of this species remains understudied compared with other Caudata (e.g. Plethodontidae) [16,17]. The fossorial habit of adult *A. maculatum* suggests that this species spends most of its time within thermally stable environments, as underground refuges provide a thermal buffer to extreme temperatures experienced aboveground [48]. Thermal biology theory predicts that active thermoregulation should not be favoured in species from thermally stable habitats, given the limited thermoregulatory benefit [43,49,50]. Thus, one may expect that fossorial species should have relatively low thermoregulatory precision, show low evidence of thermotactic behaviours [51,52], and that seasonal fluctuations in temperature should not have a relevant impact over their thermal biology [23,53]. Importantly, although *A. maculatum* is fossorial, its overall biology (e.g. reproduction, activity) follows seasonal patterns [54]. Furthermore, a recent study demonstrated that body condition in a northern population of *A. maculatum* declined in response to increasingly warmer summer and autumn temperatures over the course of 12 years of monitoring [55]. The authors also showed that *A. maculatum* experienced significant variation in underground temperatures across seasons [55]. However, the extent to which seasonality influences behavioural thermoregulation in *A. maculatum* is still unclear.

We used an integrative approach to test how seasonal acclimatization affected thermoregulatory behaviours within a thermal gradient. We first examined if salamanders showed different temporal patterns of *T*_sel_ between seasons. We tested for a position bias influencing thermoregulation by assessing whether salamanders that initially faced the cold end of the gradient would have lower *T*_sel_ compared with those that initially faced the warm end of the gradient [56]. We then tested for a relationship between cutaneous water loss and *T*_sel_ to understand if salamanders with high rates of cutaneous water loss would target relatively low *T*_sel_ [57]. We also tested whether salamanders traded increased locomotory activity for lower thermoregulatory precision. We predicted that highly mobile salamanders would sample a wider range of temperatures instead of targeting specific *T*_sel_ [41], thereby showing lower thermoregulatory precision than more stationary salamanders. Besides quantifying conventional thermoregulatory parameters [58], we also quantified *T*_sel_ skewness and kurtosis [59] to ascertain whether *A. maculatum* showed evidence of active thermoregulation. Lastly, we compared how thermoregulatory parameters differed between *A. maculatum* acclimatized to conditions that matched their active and overwintering seasons to test if salamanders showed greater evidence of thermophilic behaviours in the active season compared with the overwintering season [45].

## 2. Material and methods

### 2.1. Study site and species

Bat Lake is a fishless, naturally acidic (pH ~ 4.5−4.8), and permanent kettle lake in Algonquin Provincial Park, Ontario, Canada (45.5857° N, 78.5185° W) [60]. Bat Lake is located at an altitude of ~450 m above sea level, which is higher than the surrounding low boreal wetland region (~150 m above sea level) [61]. From a climatic standpoint, this means that *A. maculatum* living in the coniferous forest surrounding Bat Lake experience climatic conditions comparable to those found in this species’ northern range edge [55].

*Ambystoma maculatum* (Caudata: Ambystomatidae) is a medium-sized species of mole salamander that has a wide distribution in eastern North America [62]. Similar to other temperate zone ectotherms [63], activity patterns in *A. maculatum* vary seasonally and are triggered by environmental cues, such as rainfall and temperature [64]. In late winter/early spring, northern-latitude populations of *A. maculatum* emerge from underground burrows and migrate toward water bodies to breed [65]. This migratory period represents most of the aboveground activity in this species [55], although individuals may forage aboveground on relatively cool and rainy summer/autumn nights [66]; the overwintering biology of *A. maculatum* remains poorly known [65]. The approximate active period for the Bat Lake population of *A. maculatum* spans ordinal days 140−288 (20 May–17 October) [55].

On 15 May 2022, we collected 50 adult *A. maculatum* (26 females + 24 males) with the aid of a drift fence installed around the perimeter of Bat Lake as part of the Bat Lake Inventory of Spotted Salamanders (BLISS) project [67]. Logs and wood boards placed along the fence served as refuges for amphibians that reached the fence but were unable to cross it. Throughout the migratory period, researchers from BLISS conducted nighttime surveys to guarantee the timely processing and movement of amphibians. To ensure that all collected animals were at a similar physiological state [68], we only collected post-breeding individuals, which we considered to be those that were found on the lake side of the drift fence. We sexed individuals based on secondary sexual characteristics; during the breeding period, female *A. maculatum* have pleated cloacae, whereas males have papillose cloacae [69].

We only collected animals that had not been catalogued in previous BLISS monitoring efforts. When we collected an individual, we recorded its snout–vent length with a tape measure, recorded its body mass with a Pesola™ spring scale (nearest 0.1 g), and assigned it a unique identification (ID); the ID was coupled with a photograph of the dorsum of the salamander to produce an individual-level recognition system based on spot patterns. Prior to transferring the salamanders to Brock University, we allocated them into plastic containers with ventilated fitted lids (34 cm × 19.6 cm × 12 cm). We kept a total of 10 individuals per container (totalling five containers during transport); each container had sphagnum moss, pine needles and water. We placed the containers inside a transport box kept at ~4°C so that animals would not overheat or dehydrate during transportation. Prior to collecting salamanders, we obtained approval from Brock University’s Animal Care Committee (AUP 22-03-04), the Ministry of Northern Development, Mines, Natural Resources and Forestry (1100575), and Ontario Parks.

### 2.2. Husbandry

We housed salamanders in pairs within ventilated tanks that contained wet coconut husk fibre, sphagnum moss and refuges made from PVC pipes; these tanks were stacked in racks to facilitate access to animals and misted regularly to keep local humidity high. We kept the housing tanks in a facility that had temperature, humidity and photoperiod control. We changed temperature and photoperiod seasonally to mimic conditions experienced by *A. maculatum* in Bat Lake [55]. Thus, our work represents an assessment of the effects of seasonal acclimatization over thermal biology. In the spring, we kept salamanders at 7°C, 70% relative humidity (RH) and a 10 h:14 h light:dark cycle. In the summer, we kept salamanders at 14°C, 70% RH and a 14 h:12 h light:dark cycle. In the autumn, we kept salamanders at 12°C, 70% RH and a 12 h:12 h light:dark cycle. In the winter, we kept salamanders at 2°C, 70% RH and under total darkness. To transition between spring to summer conditions, we increased temperature at the rate of 1°C per day, totalling seven days necessary to go from 7 to 14°C (from 14 July 2022 to 20 July 2022). The same protocol was used to transition between summer to autumn conditions, totalling 2 days needed to go from 14 to 12°C (from 25 September 2022 to 26 September 2022). The transition between autumn to winter conditions followed a modified protocol, in which we decreased temperature at the rate of 1°C every 2.5 days, totalling 25 days necessary to go from 12 to 2°C (from 17 November 2022 to 11 December 2022). We always gave the salamanders at least four weeks to acclimatize to prevailing conditions before proceeding with our experiments. Throughout their period in the laboratory, we fed salamanders twice a week with mealworms dusted in calcium and multi-vitamin powder; water was available ad libitum. We weighed salamanders to the nearest 0.01 g every week with an analytical scale (Mettler Toledo, model PB602-S) to monitor changes in body mass as a proxy for animal wellbeing.

### 2.3. Thermal gradient set-up

**Figure 1.**
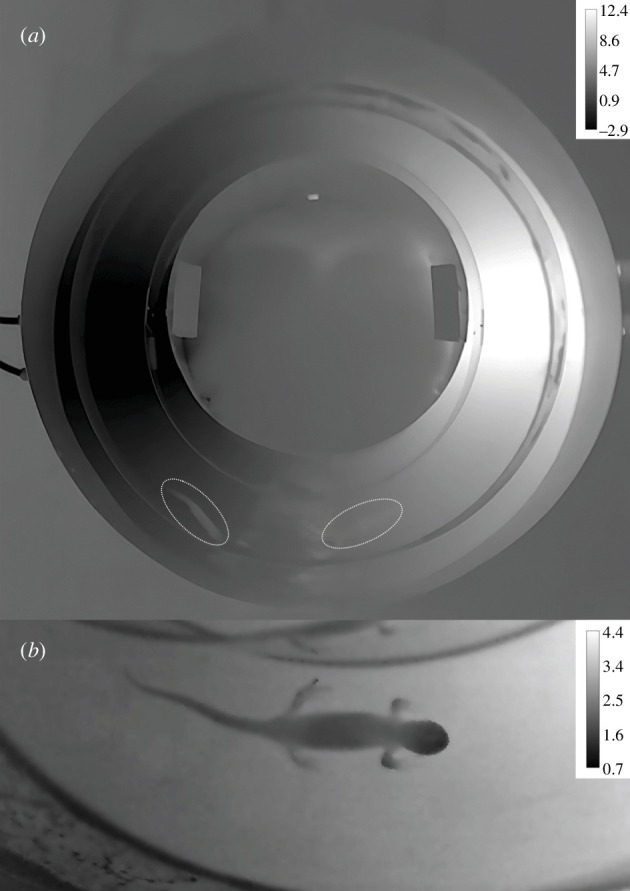
(*a*) Thermal image of the annular thermal gradient used in this study depicting the temperature range used in the overwintering season. The oval shapes denote two individuals of *Ambystoma maculatum*. The one on the lower left has a surface body temperature slightly higher than that of the floor of the thermal gradient. By contrast, the one on the lower right has a surface body temperature in equilibrium with that of the thermal gradient floor. (*b*) Thermal image of a female *A*. *maculatum* in the annular thermal gradient. Note the different temperature (°C) range bars between panels (*a*) and (*b*). In both panels, darker colours indicate lower temperatures and lighter colours indicate higher temperatures.

To assess thermoregulation, we used an annular thermal gradient (total length = 120 cm, outer radius = 45 cm, inner radius = 30 cm) built by Brock University Technical Services (figure 1*a*; electronic supplementary material, figure S1). We purposely chose an annular thermal gradient to avoid low-temperature behaviours that could be mistaken for active temperature selection (e.g. ‘corner hugging’) [38]. The floor of the annular thermal gradient had copper pipes that were connected to hot- and cold-water baths (Haake™, models DC10 and SC100, respectively), which created a near linear temperature gradient (electronic supplementary material, figure S1). For experiments conducted in the active season, the floor of the gradient ranged from approximately 3 to 25°C (range_active_ = 22°C). In the overwintering season, the floor of the gradient ranged from approximately −2.5 to 15°C (range_overwintering_ = 17.5°C). These temperature ranges represent possible habitat temperatures encountered by *A. maculatum* in Bat Lake during the active and overwintering seasons [55]. We measured temperature along the thermal gradient to the nearest 0.01°C with a thermocouple meter (Sable Systems, model TC-1000) before and after each experiment to ensure proper functioning of the gradient.

The lid of the annular thermal gradient was affixed with a 10.5 cm tall partition that allowed the simultaneous study of two individuals per experimental run (i.e. one in the inner gradient lane and another in the outer gradient lane). The lid of the thermal gradient also contained two venting holes every 90°, such that two venting holes were directly above the warm end at 0°, intermediate temperatures at 90° and 270°, and the cold end at 180°. Venting holes at 0°, 90° and 270° were connected to a water vapour bubbling system put in place to minimize hydroregulation costs within the gradient. This system consisted of a 500 ml flask filled with dechlorinated water, and kept constantly at the warmest gradient temperature depending on the thermal condition (i.e. 25°C in the active season or 15°C in the overwintering season) to ensure the highest temperatures would experience high humidity. We put a 2.5 cm aquarium air stone inside the flask; the air stone was connected to a pressure pump (HIBLOW USA, model C-5BNS-0110) through a piece of tubing so that the water in the flask could be aerated. We sealed the flask with a rubber stopper that contained two openings in it: one for the tubing connecting the pressure pump to the air stone, and another to collect water vapour generated in the flask and direct it to the thermal gradient. The pressure pump was set to aerate the water in the flask at 1000 l min^−1^, verified with a flow meter (Aalborg Instruments & Controls Inc., model PMR1-010972). The pressure created inside the flask directed water vapour from the flask to the venting holes in the gradient lid. We measured RH at different gradient temperatures with a data logger (MSR-Electronics GmbH, model MSR-145) to ensure that we had a continuous source of humid air at all gradient temperatures through the duration of our experiments. To avoid condensation due to water saturation in the gradient from limiting our ability to see the salamanders, we applied a water repellent (Stoner Invisible Glass^®^) to the interior of the thermal gradient lid before the start of each experiment. We used a paper towel to remove excess water repellent from the thermal gradient lid, so that the salamanders would not be in contact with excess liquid.

We set up the thermal gradient in the same facility where the salamanders were housed. We propped up the thermal gradient on 15 cm of insulation and foam padding to avoid building vibrations from disturbing the salamanders during experimentation. We conducted our experiments under complete darkness, matching the nocturnal habit of *A. maculatum*. We positioned two infrared illuminators (wavelength = 850 nm; TVPSii, model TP-IRBP15) 200 cm above the thermal gradient chamber to aid with visualizing the animals through a livestream. To record the behaviour and position of salamanders inside the gradient, we fixed a high-resolution webcam (Agama, model V-1325R) 180 cm above the centre of the thermal gradient. This webcam was connected to a time lapse image acquisition software (HandyAVI^®^), which recorded an image every 30 s.

### 2.4. Experimental design

The procedures described subsequently are applicable to the experiments conducted in both the active and overwintering seasons, unless otherwise stated. Importantly, in the current study, ‘active season’ pertains to salamanders acclimatized to summer conditions, and ‘overwintering season’ pertains to salamanders acclimatized to winter conditions. To measure behavioural thermoregulation, we allowed the salamanders (*n* = 50; randomly selected) a total of 8 h inside the thermal gradient (from ~20.00 to 04.00 in the active season, and from ~09.00 to 17.00 in the overwintering season). In both seasons, we gave the salamanders an initial 3 h habituation period, and used the data obtained in the subsequent 5 h (i.e. experimental period) in the analyses. We fasted salamanders for at least two weeks before experimentation to avoid thermophilic responses to feeding [70]. We tested two individuals at a time, choosing at random whether a salamander would be tested in the inner or outer gradient lane. We always allowed a minimum interval of 12 h between trials after disinfecting the stage of the thermal gradient with 70% ethanol at the end of a trial.

Before introducing salamanders into the gradient, we placed them individually into a container filled with 30 ml of dechlorinated water for 15 min, so water could be absorbed through the skin; we always handled the animals using nitrile gloves. Following this, we weighed the salamanders and placed them into the gradient, determining at random whether salamanders would be initially facing the cold or warm end of the gradient. For experiments done in the active season, we introduced the salamanders into the gradient at the 14°C point, so that the first temperature they encountered therein would match their prevailing thermal condition. We followed the same protocol in the overwintering season, but at the 2°C point. To minimize handling stress on thermoregulatory behaviours, we did not handle or manipulate the salamanders during the experiments.

We verified that thermoregulatory differences (i.e. median *T*_sel_) between the active and overwintering seasons were not the outcome of offering different temperature ranges (i.e. range control) in the thermal gradient between seasons or conducting our experiments during a different time of day (i.e. time-of-day control). To this end, we tested a subset of winter-acclimatized salamanders (*n* = 20) using a thermal gradient that ranged from approximately −2.5 to 19.5°C (hereafter ‘range control’; range_control_ = 22°C, similar in span to the summer), and another subset of winter-acclimatized salamanders (*n* = 20) using the overwintering thermal gradient range from ~20.00 to 04.00 (hereafter ‘time-of-day control’, conducted at the same time of day as the summer). If the effect of acclimatization over behaviour is consistent, then there should be no differences between the overwintering experiments and range control, or between the overwintering experiments and time-of-day control. After finishing the experiments, we removed the salamanders from the thermal gradient and measured their body mass. We used the difference in body mass before and after experiments as a proxy of cutaneous water loss during the 8 h of experimentation. After weighing, we placed the salamanders back into their housing tanks.

### 2.5. Calculating *T*_sel_

To calculate *T*_sel_, we captured a total of 960 images over the course of each 8 h long experiment, and imported the entire image sequence into Fiji [71]. From each image sequence, we recorded when the experiment was conducted (i.e. active or overwintering season), which individual was being tested in the inner and outer lanes, the sex of the individuals and whether the salamanders were placed in the gradient facing the cold or warm end. We manually tracked the location of the salamanders in the thermal gradient for each image in the sequence using the manual tracking plug-in in Fiji [71]. For each image sequence, we obtained a set of Cartesian (*x*, *y*) coordinates that had *x*, *y* = 0, 0 as the lower left of the image. We then used a series of calculations to convert salamander body position into selected gradient temperatures. Given that the centre of our images did not correspond to the centre of the thermal gradient (i.e. centroid), we subtracted the Cartesian coordinates of the centroid from those of the salamander’s position in the thermal gradient:


$$\left(x^{\prime },y^{\prime }\right)={Salamander}_{\left(x,y\right)}-{Centroid}_{\left(x,y\right)},$$

where (*x*′, *y*′) represent normalized Cartesian coordinates. We converted these normalized Cartesian coordinates into distance by multiplying (*x*′, *y*′) values by 0.0010929 m pixel^−1^; this conversion was determined empirically based on known gradient dimensions (see [41]). We then converted normalized Cartesian coordinates into polar coordinates (*r*, *θ*). Here, *r* is the hypotenuse determined by the *x*′ and *y*′ coordinates, and *θ* is the reciprocal of the *y*′/*x*′ tangent noted as degrees around a circle. For positive *y*′ values, *θ* ranged from 0° to 180°, and for negative *y*′ values, *θ* ranged from 0° to −180°. Given that the annular thermal gradient was symmetrical along the 0° horizontal axis, we converted *θ* values to an absolute value of |*θ|*. Lastly, we converted the final corrected *θ* position (*θ*′) into temperature. To do so, we used a second-order regression equation that described changes in temperature between known angular positions within the thermal gradient. We empirically measured these changes in temperature along the gradient in all experiments.

We assumed that the body temperature of the salamanders was in thermal equilibrium with the temperature of the gradient floor. Research in lizards showed that *T*_sel_ matched core *T*_b_ over long time courses [72]. Given the small body mass of the individuals used in our study (body mass range: 5.05−26.74 g) and the relatively long length of our experiments, we considered that a short time span would be necessary for salamander *T*_b_ to equilibrate with gradient floor temperature. To verify this, we used a thermal camera (FLIR Systems, model FLIR T1030) to visually assess if the surface body temperature of an individual salamander matched the temperature of the thermal gradient. Assuming an object emissivity of 0.95 [73], we processed the thermograph with ThermimageJ [74] plugins for Fiji that were verified against algorithms in FLIR Research Studio software. We adjusted camera calibration constants and object parameters (e.g. RH, object distance) according to conditions measured in the laboratory at the moment of thermal image sampling. To obtain measurements of dorsal mid-body (*T*_midbody_) and snout (*T*_snout_) skin temperature, we digitally drew a region of interest (ROI) over the salamander. We also drew an ROI over the thermal gradient floor immediately adjacent to the animal to obtain a measurement of gradient floor temperature (*T*_gradient_). From each ROI, we obtained an average temperature. We detected evidence of regional heterothermy [75] in the salamander, with the mid-point of the body showing average temperature values closest to those of the gradient floor compared to the snout (*T*_gradient_ = 3.16°C; *T*_midbody_ = 2.46°C; *T*_snout_ = 2.23°C) (figure 1*b*). As such, we manually tracked the salamanders based on the position of the mid-point of their bodies, and used these values to calculate thermal set-point parameters [58].

Following image analysis, we partitioned each salamander’s surface body temperatures into percentiles. The 25th percentile denotes the lower *T*_sel_ bound, the 50th percentile denotes the median *T*_sel_, and the 75th percentile denotes the upper *T*_sel_ bound. The difference between the upper and lower *T*_sel_ bounds is the *T*_sel_ range. Ectotherms that thermoregulate within narrow *T*_sel_ ranges are considered to be precise thermoregulators [76]. In the context of seasonality, seasonal changes in *T*_sel_ parameters are an indication of a species’ potential for plasticity in temperature-dependent traits [23]. To obtain a measure of activity in the gradient, we quantified the distance moved (*d*_*t*_) by each salamander every 30 s during the length of our experiments. Given that we used an annular thermal gradient, *d*_*t*_ follows the distance described by the arc of a circle:


$$d_{t}=\frac{0.5\times \left(r_{t}+\;r_{t-1}\right)\times \pi \times 2\times |\theta_{t}-\theta_{t-1}|}{360},$$

where *r* is the mean radius, *θ* are polar coordinates, and *t* and *t −* 1 are adjacent time points for all *n* time points. To determine the total distance moved by each individual, we took the sum of *d*_*t*_:


$$D_{t}=\sum_{1}^{n-1}d_{t},$$

where *n* is the total number of images (*n* = 960).

### 2.6. Data analysis

We performed all analyses using RStudio (v. 2023.06.2) in R (v. 4.2.2) [77] with a significance level of 0.05. We visually inspected Q–Q and P–P plots in search for deviations from normality and homoscedasticity using the ‘fitdist’ function from the fitdistrplus package [78]. We built generalized linear models (GLMs) with the ‘glm’ function from the stats package [77], linear mixed-effects models (LMMs) with the ‘lmer’ function from the lme4 package [79] and generalized linear mixed-effects models (GLMMs) using the function ‘glmer’ from the lme4 package [79]. To assess residual autocorrelation, we used the ‘checkresiduals’ function from the forecast package [80], and the ‘qqnorm’ and ‘acf’ functions from the stats package [77]. We assessed model fit with the ‘check_model’ function from the performance package [81]. To visualize fixed model effects, we used the ‘plot’ function (graphics package [77]) to plot an object created with the ‘allEffects’ function from the effects package [82].

To compare if *T*_sel_ distribution parameters differed from zero, we used a one sample *t*‐test fit with the ‘t.test’ function from the stats package [77]. We also assessed the kurtosis of our individual-level *T*_sel_ data through a Jarque–Bera goodness of fit test using the ‘col_jarquebera’ function from the matrixTests package [83]. If kurtosis <3, then the *T*_sel_ distribution is platykurtic. If kurtosis >3, then the *T*_sel_ distribution is leptokurtic. From a biological perspective, a platykurtic *T*_sel_ distribution would indicate that salamanders are able to tolerate a wide range of gradient temperatures, whereas a leptokurtic *T*_sel_ distribution would point to salamanders targeting a narrow range of gradient temperatures. We performed Bonferroni correction of *p*-values when multiple models were fitted on data derived from the same subset of experiments through the ‘p.adjust’ function from the stats package [77]. Finally, we created figures using the ggplot2 [84], cowplot [85] and patchwork [86] packages. We provide details on the models we used to test our hypotheses, R functions and packages in electronic supplementary material, table S1; our data and code can be accessed from the Brock University Dataverse [87].

## 3. Results

### 3.1. Temporal trends in *T*_sel_ and validation of experimental assumptions

**Figure 2.**
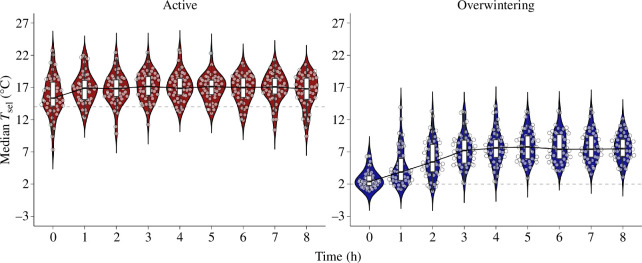
Temporal variation in the selected body temperature (*T*_sel_) of *Ambystoma maculatum* throughout 8 h during the active and overwintering seasons. In both panels, the grey dashed lines represent the acclimatization temperatures in the active (14°C) and overwintering (2°C) seasons. Boxes denote the among-individual 25th and 75th bounds of *T*_sel_ at a given hour. The horizonal line in each box indicates the median *T*_sel_, and the black line connecting each median *T*_sel_ showcases the hourly change in this parameter. Temperatures selected by each individual salamander are shown as semi-transparent dots. The violin plots show the kernel density probability of *T*_sel_ data at different hours of experimentation.

In both seasons, we found that *T*_sel_ varied over the 8 h of experiment (active: χ^2^_(8)_ = 19.60, *p* = 0.01; overwintering: χ^2^_(8)_ = 334.03, *p* = 2.2 × 10^−16^). Median *T*_sel_ values were offset higher than prevailing thermal conditions in both seasons (figure 2). Median *T*_sel_ did not differ between the inner and outer gradient lanes in either the active (χ^2^_(1)_ = 0.46, *p* = 0.49) or overwintering seasons (χ^2^_(1)_ = 0.67, *p* = 0.41). Total distance moved was similar between lanes in the active (χ^2^_(1)_ = 0.10, *p* = 0.76) and overwintering seasons (χ^2^_(1)_ = 0.01, *p* = 0.94). Body mass differed between the beginning and the end of the experiments (active: χ^2^_(1)_ = 168.51, *p* < 2.2 × 10^−16^; overwintering: χ^2^_(1)_ = 77.26, *p* < 2.2 × 10^−16^). Salamanders lost, on average, 0.15 g h^−1^ in the active season compared to 0.04 g h^−1^ in the overwintering season during the 8 h long experiments. Despite this, body mass loss was not influenced by median *T*_sel_, distance moved or sex within either season (electronic supplementary material, table S2). Our water vapour bubbling system kept high RH values at all gradient temperatures ranging from −2.5 to 25°C (average ± s.d. = 92.93 ± 6.47% RH).

### 3.2. Behavioural thermoregulation parameters

**Figure 3.**
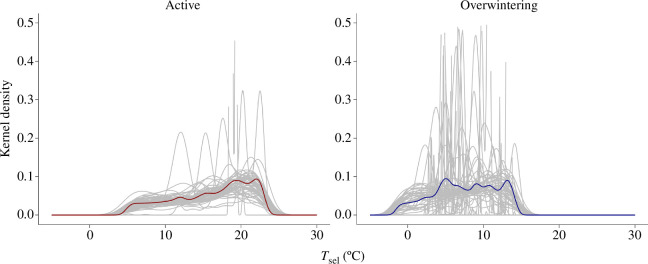
Kernel density estimations of median selected temperature (*T*_sel_) highlighting the negatively skewed and platykurtic nature of *T*_sel_ in *Ambystoma maculatum*. In both panels, the grey lines depict the *T*_sel_ distribution of each individual, whereas the colour-coded lines show the mean *T*_sel_ distribution considering all individuals tested within a season (*N* = 50 each season). Values above 0.5 on the *y*-axis pertain to salamanders that remained mostly stationary during the experiments.

**Figure 4.**
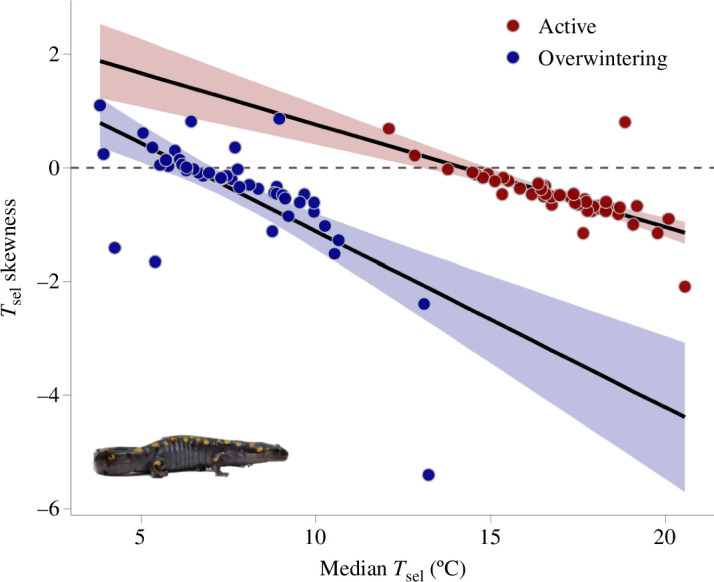
Relationship between skewness and median selected temperatures (*T*_sel_) for *Ambystoma maculatum*. The solid black lines and coloured fills indicate the line of best fit and the corresponding 95% confidence interval, respectively. The grey dashed line indicates the zero. Dots represent individual *A*. *maculatum* (*N* = 50 per season). The inset shows a female *A*. *maculatum*.

We used the experimental period to calculate the median, lower and upper *T*_sel_ bounds from individual salamander *T*_sel_ distributions (table 1) (electronic supplementary material, figure S2). Whether salamanders were initially facing the cold or warm end of the gradient had no effect over median *T*_sel_ in either season; sex and body mass also did not affect median *T*_sel_ (electronic supplementary material, table S3). In both seasons, we found that the more an individual moved, the wider its *T*_sel_ range. Neither sex nor body mass mediated this effect (table 2). In the active season, the individual distribution of *T*_sel_ was negatively skewed and different from a normal distribution (*T*_sel_ skewness_active_ = −0.50; *t* = −8.24, *p* = 8.28 × 10^−11^). *T*_sel_ was platykurtic in the active season (kurtosis = 2.15) (figure 3); we obtained similar results for skewness (*T*_sel_ skewness_overwintering_ = −0.38; *t* = −2.74, *p* = 0.01) and kurtosis (kurtosis = 2.07) in the overwintering season (figure 3). In both seasons, individuals with higher median *T*_sel_ showed more negative *T*_sel_ skew (*β*_active_ = −0.20; *t* = −7.41, *p* = 2 × 10^−9^; *β*_overwintering_ = −0.30; *t* = −6.22, *p* = 1.14 × 10^−7^; figure 4). All thermoregulatory parameters studied herein differed between seasons, except for *T*_sel_ skewness and total distance moved, with no effect of sex or body mass (table 1). We did not find thermoregulatory differences between animals tested in the overwintering season and the range control, except for *T*_sel_ range and total distance moved (electronic supplementary material, table S4). We obtained the same results when comparing salamanders tested in the overwintering season and the time-of-day control (electronic supplementary material, table S5).

**Table 1.** Parameters associated with temperature selection (*T*_sel_) for *Ambystoma maculatum* studied in the active and overwintering seasons. *p*-values in bold denote significant differences in a given trait between the active and overwintering seasons. s.d., standard deviation; min, minimum value; max, maximum value; χ^2^, chi-square.

	active (*n* = 50)	overwintering (*n* = 50)	comparisons		
median *T*_sel_ (°C)					
mean ± s.d.	17.0 0 ± 1.77	7.61 ± 2.10	χ^2^_(1)_ = 734.00, ***p*** **< 0.001**		
median (min, max)	17.40 (12.10, 20.60)	7.37 (3.83, 13.20)			
lower *T*_sel_ bound (°C)					
mean ± s.d.	12.60 ± 2.11	4.89 ± 2.60	χ^2^_(1)_ = 263.42, ***p*** **< 0.001**		
median (min, max)	12.40 (8.22, 20.10)	4.46 (1.78, 13.20)			
upper *T*_sel_ bound (°C)					
mean ± s.d.	20.10 ± 1.15	10.40 ± 2.00	χ^2^_(1)_ = 1022.76, ***p*** **< 0.001**		
median (min, max)	20.30 (17.20, 22.40)	10.80 (4.49, 14.10)			
*T*_sel_ range (°C)					
mean ± s.d.	7.48 ± 1.91	5.53 ± 2.65	χ^2^_(1)_ = 18.19, ***p*** **< 0.001**		
median (min, max)	7.85 (0.80, 11.20)	6.18 (0.07, 9.16)			
*T*_sel_ skewness					
mean ± s.d.	−0.51 ± 0.44	−0.38 ± 0.97	χ^2^_(1)_ = 0.81, *p* = 0.36		
median (min, max)	−0.53 (−2.09, 0.80)	−0.19 (−5.41, 1.10)			
total distance moved (m)					
mean ± s.d.	109.00 ± 67.10	78.20 ± 70.50	χ^2^_(1)_ = 7.10, *p* = 0.054		
median (min, max)	95.20 (0.26, 336)	58.00 (0.14, 262)			
body mass loss (%)					
mean ± s.d.	8.68 ± 3.40	2.66 ± 1.84	χ^2^_(1)_ = 49 722.03, ***p*** **< 0.001**		
median (min, max)	8.95 (0.20, 14.7)	2.18 (0.11, 7.37)			

We made comparisons between seasons using linear mixed effects models that had season, body mass, and sex as the predictor variables, as well as salamander ID as a random term. Given that neither body mass nor sex mediated differences between seasons, we only report χ^2^ and *p*-values for season in each model.

**Table 2.** Parameter estimates (*β*), 95% confidence intervals (95% CI), and *p*-values for the models assessing how total distanced moved affected thermoregulatory precision in *Ambystoma maculatum*. The models had *T*_sel_ range as the response variable; distance moved, body mass, and sex as the predictor variables. Significant parameters are denoted in bold.

active			
predictors	*β*	95% CI	*p*
intercept	7.91	7.22–8.61	**<0.001**
total distance moved	0.02	0.01–0.02	**<0.001**
sex (male)	0.05	−0.10–0.21	0.492
body mass	−0.89	−2.02–0.23	0.120
observations	50		
*R* ^2^	0.368		

overwintering			
predictors	*β*	95% CI	*p*
intercept	5.18	4.40–5.97	**<0.001**
total distance moved	0.03	0.02–0.04	**<0.001**
sex (male)	0.03	−0.12–0.18	0.679
body mass	0.73	−0.56–2.02	0.270
observations	50		
*R* ^2^	0.590		

## 4. Discussion

Theory predicts relaxed selection pressure over thermoregulation in ectotherms from thermally stable habitats, like underground environments [43,50]. As a result, fossorial ectotherms typically have low *T*_sel_, low thermoregulatory precision, and show low evidence of thermophilic behaviours compared to species from thermally variable habitats [51,52]. While these patterns are well established in reptiles, we still have a limited understanding of amphibian thermoregulation in the context of fossoriality. By comparing behavioural thermoregulation parameters between seasons, we demonstrated that *A. maculatum* engages in active thermoregulation despite its fossorial habit. Thermoregulation differed between seasons, with salamanders selecting higher gradient temperatures and showing greater evidence of thermophily in the active relative to the overwintering season. Our work also gives support to experimental assumptions commonly made but rarely tested in thermal biology studies. Together, our findings illustrate that the integration of behavioural and thermal biology measurements is a prominent means to further the knowledge about the maintenance of thermal balance in amphibians.

### 4.1. Inferences of behavioural thermoregulation

We found that *T*_sel_ varied over the course of our 8 h long experiments, which was consistent with studies evaluating hourly changes in the *T*_sel_ of amphibians [37,88]. Although daily shifts in *T*_sel_ may be regulated by endogenous aspects [89], the pattern we observed is likely explained by animals gaining a spatial awareness of the thermal gradient [72] and recovering from stress caused by manipulation [90] in the initial hours of the experiment. Our study also validated assumptions relevant to the design of behavioural thermoregulation experiments. Measuring the *T*_sel_ of multiple individuals at a time is common practice in thermal ecology research [91,92]. Researchers typically introduce animals into separate gradient lanes and assume that thermoregulation is not affected by experimental design. This, however, is rarely verified [16], and overlooks the possibility of animals altering their thermoregulatory behaviour from detecting the presence of nearby organisms. For example, *Amalosia lesueurii* (Squamata: Diplodactylidae) were shown to avoid thermally suitable areas after recognizing the scent of potential predators [93]. Moreover, *Salamandra salamandra* (Caudata: Salamandridae) were shown to be capable of sex discrimination through olfaction, which resulted in individuals changing their spatial distribution after detecting the scent of a conspecific [94]. Our current understanding of how sensory stimuli (e.g. olfactory, acoustic, magnetic, mechanic) may interact with realized thermoregulation is still limited and warrants further research.

Work in the fossorial *Lialis burtonis* (Squamata: Pygopodidae) suggested that lizards would end up with relatively low *T*_sel_ if introduced into the cold end of a thermal gradient; the opposite was true when lizards were introduced into the warm end [56]. These data suggested that *L. burtonis* tolerated rather than actively selected gradient temperatures, which corroborated the expectation of low evidence of thermotactic behaviours in fossorial ectotherms. In our study, we controlled for the location at which *A. maculatum* were first placed in the thermal gradient by always introducing them at the temperature that corresponded to the prevailing acclimatization regime. The initial direction *A. maculatum* faced when introduced into the thermal gradient did not impact median *T*_sel_ in either season. Thermal gradient design could explain why a position bias over *T*_sel_ was observed in *L. burtonis* but not in *A. maculatum* despite both species being fossorial. Indeed, *L. burtonis* were tested in a rectangular gradient [56], whereas we tested *A. maculatum* in an annular thermal gradient. Rectangular thermal gradients are the most common gradient type in thermal biology studies (e.g. [15,38,95]). However, the rectangular shape may produce a site-specific bias towards ends or corners of the thermal gradient, resulting in misleading inferences of *T*_sel_ [96]. Although less used in thermal biology research (but see [76,97,98]), annular thermal gradients are argued to allow for easier navigation [41] and to encourage animals to actively select gradient temperatures [96], thereby facilitating assessments of thermotaxis and the relationship between locomotion and *T*_sel_. Importantly, formal assessments of whether the use of rectangular and annular gradients differ in the ability to infer thermoregulation have yet to be made in terrestrial ectotherms.

Body mass differed between the start and the end of the experiments in both seasons; however, body mass loss was not explained by median *T*_sel_, total distance moved, or sex. Thus, salamanders with relatively high body mass loss did not move toward cooler areas of the gradient as a primary defence mechanism against possible desiccation [57]. This suggests that we effectively minimized hydroregulatory costs within the thermal gradient, and that our experimental conditions did not compromise hydration status, which is critical for amphibian thermoregulation [99,100]. Indeed, the lowest RH value we recorded in the thermal gradient was ~80% at 12°C in the active season. While information on soil moisture preference is unavailable for *A. maculatum*, the congener *A. opacum* preferred soils of 74% RH at 13°C [101]. Coupled with the fact that we never observed our study individuals employing hydroregulatory behaviours (e.g. coiling) [102], these results suggest that our behavioural observations were thermoregulatory in nature. Despite this, amphibians are often faced with the risk of dehydration, especially at high environmental temperatures. For example, studies have shown that an interaction between temperature and dehydration explains interspecific differences in locomotion in toads [7], and that the effect of seasonality over hydroregulatory and thermoregulatory parameters is trait-dependent in frogs [19]. In salamanders, temperature was shown to be an important factor behind desiccation resistance [103], with implications for the duration of activity [104]. In Ambystomatidae, most of the work to date has focused on determining interspecific differences in dehydration rates or assessing the effect of thermal stress on the maintenance of water balance [105–107]. To further understand the trade-off between thermoregulation and hydroregulation in salamanders, future work may assess whether *A. maculatum* uses behaviour to defend a preferred RH range once thermoregulatory costs are minimized.

### 4.2. Seasonal acclimatization of behavioural thermoregulation

Salamanders traded-off increased locomotion for lower thermoregulatory precision, as evidenced by a positive relationship between *T*_sel_ range and total distance moved. This effect was consistent in salamanders tested in both seasons, and matches a pattern previously reported in the semi-fossorial *Storeria occipitomaculata* (Squamata: Colubridae) [41]. Additionally, this trade-off was not mediated by sex or body mass in either season, suggesting a possible link between activity levels and thermoregulation irrespective of sexual size dimorphism [108]. Importantly, movement within a laboratory thermal gradient is primarily associated with active temperature selection. In nature, however, movement may depend on factors such as foraging tactics, the presence of nearby predators, and climate [109–111], all of which may conflict with an ectotherm’s motivation to thermoregulate [112]. A recent mark–recapture study suggested a link between minimum temperatures and increased movement in *A. maculatum*, *A. jeffersonianum* and *A. laterale-jeffersonianum* [113]. However, the interplay between salamander movement and thermoregulation in natural settings remains unclear. By combining laboratory inferences of thermoregulation with field measurements of *T*_b_ and radio-tracking (e.g. [24 114,114]), one may clarify whether behavioural thermoregulation is relevant to salamanders in nature.

Our data on *T*_sel_ skewness and kurtosis supported the prediction that ectotherms should be more tolerant of low than high temperatures [115]. Indeed, in both seasons, we found individual *T*_sel_ to be negatively skewed and platykurtic, which fits the characteristics of thermal performance curves, especially in thermal generalists [29,116]. We also found that salamanders with higher *T*_sel_ showed a more pronounced negative *T*_sel_ skew than those with lower *T*_sel_ in both seasons. The reasons behind the ubiquity of the negative skew in ectotherm *T*_b_ and *T*_sel_ are likely numerous (see [116]). From a thermosensory standpoint, warm-sensitive neurons are more responsive than cold-sensitive ones [117], which may result in enhanced sensory-driven thermal behaviours at relatively warm temperatures. Alternatively, the negative skew in *T*_sel_ could be a product of the exponential relationship between *T*_b_ and physiological performance [59], as often observed in thermal performance curves [115]. In ectotherms, performance typically rises slowly with temperature until it reaches a maximum value, and then drops sharply [118]. Thermal performance curves have several uses (e.g. [119,120]), including providing a framework to understand the evolution of thermal specialists and generalists [121]. Ectotherms from stable habitats tend to be thermal specialists, which are those that have highly asymmetric and narrow thermal performance curves [29]. Conversely, ectotherms from highly seasonal habitats tend to be thermal generalists, which are animals that trade-off performance peak for a wide performance breadth [29]. Such species often show seasonal lability in thermal biology traits, which enhances the maintenance of thermal balance across seasons [23]. Quantifying seasonal changes in performance traits should clarify if *A. maculatum* is a thermal generalist and further our knowledge of the possible drivers of the negative *T*_sel_ skew in ectotherms.

Our results supported the presence of active thermoregulation in *A. maculatum*, highlighting that seasonal acclimatization prompted a shift in *T*_sel_ and associated thermotactic behaviours. However, thermoregulation in *A. maculatum* may not be as precise as in species that live primarily aboveground. Indeed, *A. maculatum* consistently thermoregulated between relatively wide *T*_sel_ ranges. The *T*_sel_ range value we obtained in the active season is comparable to that of the fossorial *Saiphos equalis* (Squamata: Scincidae), which had a mean *T*_sel_ range of 7°C [53]. Our *T*_sel_ values are also consistent with the range of values reported in laboratory studies of amphibian *T*_sel_ (reviewed in [35]). For instance, our *T*_sel_ measurements in the active season are similar to those of summer-caught *A. tigrinum* (mean ± s.d.; *T*_sel_ = 18.4 ± 0.6°C), a fossorial congener of comparable body size [37]. We also found seasonal differences in all thermoregulatory parameters except for *T*_sel_ skewness. These results are supported by previous research that indicated a heightened acclimatory capacity of *T*_sel_ and critical thermal maximum in *A. maculatum* relative to *A. texanum* [122]. We found that *A. maculatum* thermoregulated more precisely in the overwintering than in the active season. This is a recurrent pattern in lizards, typically interpreted as increased energetic allocation toward thermoregulation in the face of adverse climatic conditions [38,123]. In our study, however, increased thermoregulatory precision in the overwintering season may be explained by an effect of seasonal acclimatization over locomotion. Winter-acclimatized *A. maculatum* decreased their locomotory activity compared to the experiments conducted in the summer, which may have translated into *A. maculatum* thermoregulating between a narrower *T*_sel_ range in the overwintering compared to the active season. This observation may also explain why the *T*_sel_ range and total distance moved differed between the range control and overwintering experiments, as well as why the *T*_sel_ range and total distance moved differed between the time-of-day control and overwintering experiments.

## 5. Conclusions

The cryptic nature of many fossorial ectotherms has limited research into their physiology and behaviour [124]. This resulted not only in knowledge gaps about their basic biology but also in an overreliance on untested assumptions about the role of fossoriality in shaping the physiology and behaviour of ectotherms [125]. Our study highlights how seasonal acclimatization affects the fossorial *A. maculatum* through seasonal changes in thermal biology. Our work challenges the expectation of low evidence of thermophilic behaviours in fossorial species by showing *T*_sel_ values to be offset higher than prevailing thermal conditions in both the active and overwintering seasons. Moreover, our finding of a 9.4°C shift in *T*_sel_ between seasons adds to evidence in support of seasonal plasticity in thermal traits (e.g. [23,32]), and against the use of the ‘final thermal preferendum’ concept as a paradigm in ectotherm thermal biology [126]. We emphasize the importance of integrating behavioural observations with measurements of thermal biology traits to better understand the mechanisms underlying *T*_b_ control in amphibians [15]. We also highlight that considering the influence of seasonality over thermal biology should contribute to the goal of elucidating behavioural responses to environmental variability in ectotherms [23]. Ultimately, our findings are aligned with work showing that temperature seasonality is a relevant aspect explaining a long-term shift in body size and condition in *A. maculatum* [55]. Given that thermal biology underpins most organismal processes in ectotherms, an inability to realize *T*_sel_ in the field could translate into salamanders being unable to maximize energy turnover and thus explain why *A. maculatum* are getting smaller over time with warming climates [55]. In this context, future work may undertake an energetic perspective [127], and assess how energy metabolism varies in response to seasonal shifts in temperature.

## Data Availability

The data and code can be accessed from the Brock University Dataverse [87]. Electronic supplementary material is available online [128].
